# Covalent Organic Frameworks as Single-Site Photocatalysts for Solar-to-Fuel Conversion

**DOI:** 10.1021/jacs.3c11539

**Published:** 2024-03-28

**Authors:** Liang Yao, Alexander M. Pütz, Hugo Vignolo-González, Bettina V. Lotsch

**Affiliations:** † Max Planck Institute for Solid State Research, Heisenbergstrasse 1, 70569 Stuttgart, Germany; ‡ Department of Chemistry, University of Munich (LMU), Butenandtstrasse 5-13, 81377 Munich, Germany; § E-Conversion and Center for Nanoscience, Lichtenbergstraße 4a, Garching, 85748 Munich, Germany

## Abstract

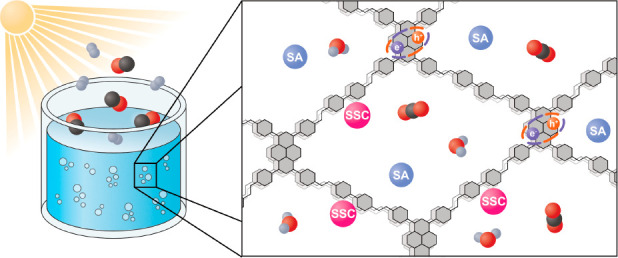

Single-site photocatalysts (SSPCs) are well-established as potent platforms for designing innovative materials to accomplish direct solar-to-fuel conversion. Compared to classical inorganic porous materials, such as zeolites and silica, covalent organic frameworks (COFs)—an emerging class of porous polymers that combine high surface areas, structural diversity, and chemical stability—are attractive candidates for SSPCs due to their molecular-level precision and intrinsic light harvesting ability, both amenable to structural engineering. In this Perspective, we summarize the design concepts and state-of-the-art strategies for the construction of COF SSPCs, and we review the development of COF SSPCs and their applications in solar-to-fuel conversion from their inception. Underlying pitfalls concerning photocatalytic characterization are discussed, and perspectives for the future development of this burgeoning field are given.

## Introduction

Given the urgency of alleviating the significant anthropogenic impact on climate change, the demand for replacement of fossil fuels with clean and renewable energy carriers is increasing. Due to the massive abundance of solar energy, directly converting sunlight to fuels in the form of hydrogen and other high-energy carbon compounds (CO, CH_3_OH, HCOOH, etc.), commonly called “solar fuels”, has been considered as one of the most promising alternatives for fossil fuels.^1−3^ Among the various emerging approaches for solar fuel production, such as photovoltaic-powered electrolysis and photoelectrochemical devices, photocatalytic systems tend to be the most scalable due to their simplified design, and are predicted to provide the lowest operational cost with a similar solar-to-fuel conversion efficiency and lifetime, based on techno-economic analysis.^4−6^ Therefore, in the past decades, enormous efforts have been devoted to material design and technology development for producing solar fuels via a photocatalytic process.

Photocatalytic production of solar fuels is a multistep process that employs photogenerated charges, obtained by illuminating semiconductor materials, to accomplish fuel-forming reactions at catalytically active sites. The efficiency of a photocatalytic system is given as the product of the efficiencies of the individual steps from charge carrier generation to the catalytic turnover: ϕ = ϕ_gen_ × ϕ_sep_ × ϕ_trans_ × ϕ_cat_, where ϕ_gen_ is the efficiency of the semiconductor generating electron–hole pairs upon illumination, ϕ_sep_ the efficiency of charge carrier separation/exciton dissociation, ϕ_trans_ the efficiency of charge carrier transport to the semiconductor surface, and ϕ_cat_ the efficiency of the catalytic reaction.^7^ Hence, while the photocatalytic performance of a photocatalyst strongly relies on the intrinsic properties of the semiconductor, modulating the catalytic efficiency of the active sites has a substantial influence on the photocatalytic activity as well.^8^ Indeed, since Taylor introduced the term of active site in the general field of catalysis in 1925, considerable efforts were directed toward engineering the properties of catalytically active sites, and several innovative catalyst design strategies have been proposed to obtain high catalytic efficiency while simultaneously reducing the usage of catalytic species.^9−12^ Among them, single-site catalysts possess the compelling characteristics of well-defined structures and uniform, spatially separated active sites featuring identical energies of interaction with the reactants. The concept of single-site catalysts has evolved into a powerful tool and burgeoning field ever since its inception in 1990s.^10,13,14^ Introducing single-site catalysts to photocatalysts for constructing single-site photocatalysts (SSPCs) has been recognized as an effective strategy to enhance the utilization of catalytically active atoms as well as the activity and selectivity of photocatalysts.^15,16^ Technically, single-site catalysts can be homogeneous or heterogeneous,^17^ depending on whether the single-site catalyst exists in the same phase or different phases with the reactant or product; therefore, both homogeneous and heterogeneous single-site catalysts can be used for constructing SSPCs. Although homogeneous catalysts generally could display a higher atom utilization efficiency, heterogeneous catalysts have the advantages of easy separation and recycling, as well as increased operational stability, and are consequently preferred for industrial applications.^17^ As defined by Thomas and co-workers, the active sites of single-site heterogeneous catalysts include the form of atoms, ions, molecular complexes and clusters, while all active sites are catalytically identical.^10^ Since porous materials provide considerably enlarged surface areas, incorporating single-site catalysts into porous scaffolds or porous photoabsorbers helps to improve the utilization of photogenerated charges. Thus, various porous supports, such as zeolites,^18^ mesoporous silicas,^18^ and metal organic frameworks,^19,20^ have been widely applied to develop SSPCs.

**Figure 1:**
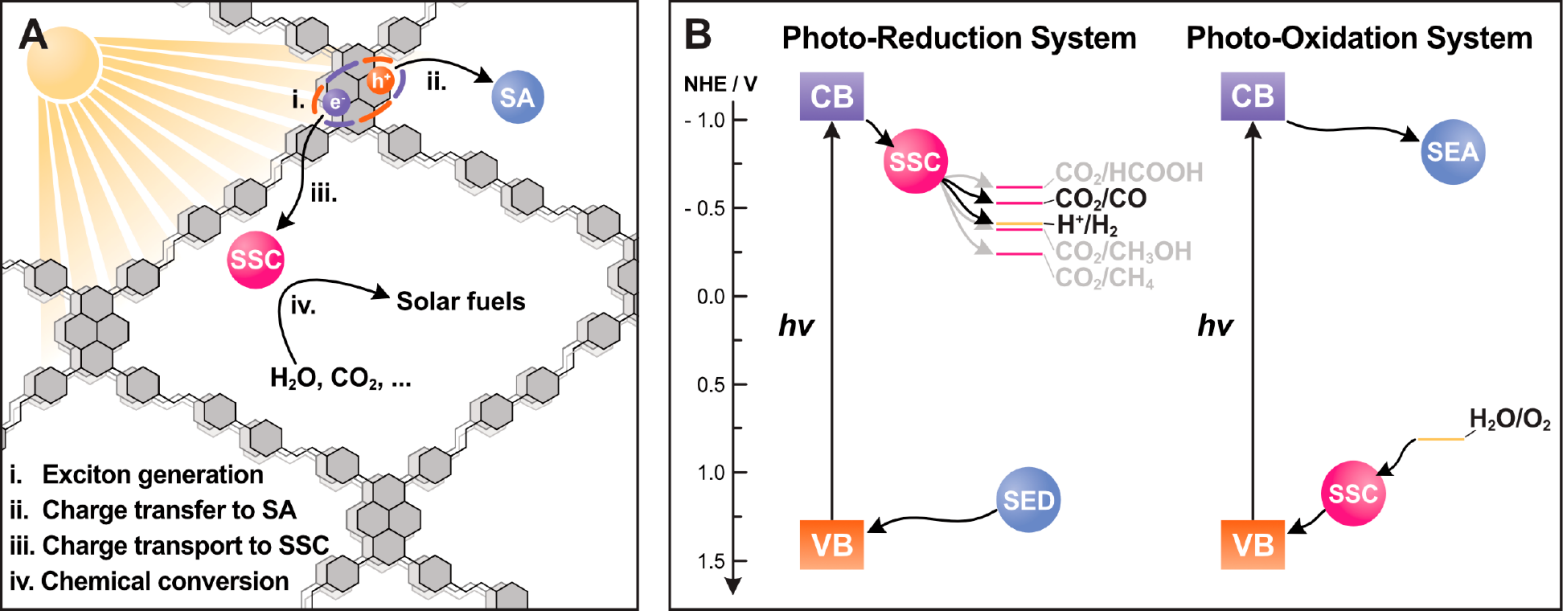
(A) Schematic illustration of the main processes for solar-to-fuel conversion with COFs as single-site photocatalysts. (B) Energetic requirements for the COFs applied to photocatalytic solar-to-fuel conversion and the equilibrium potentials versus NHE (pH 7) of the reduction and oxidation half-reactions. SSC, single-site catalyst; SA, sacrificial agent; SEA, sacrificial electron acceptor; SED, sacrificial electron donor; CB, conduction band; VB, valence band.

Covalent organic frameworks (COFs) are a class of porous and crystalline organic polymers, which combine significant diversity and regularity in their chemical structures and functionalities.^21,22^ By deliberately selecting suitable building blocks and linkages, COFs can be designed to harvest visible light while showing excellent stability under various photocatalytic operational conditions. In the past years, COFs have emerged as a new powerful platform for SSPCs (Figure 1A). In this Perspective, we present an overview of COF SSPCs for solar fuel production, including the general concepts of COF SSPCs and the strategies used to construct COF SSPCs. The applications of COF SSPCs in photocatalytic water splitting and CO_2_ conversion are highlighted. With the aim of promoting the development of the field, the underlying pitfalls and future opportunities of COF SSPCs are also discussed.

## General Concepts of COF SSPC Design

To successfully implement the fuel-forming reaction and achieve a high solar conversion efficiency, both the thermodynamics of the reaction and the kinetics associated with forming and breaking chemical bonds are crucial (Figure 1B). First, the selected COF photocatalysts should possess thermodynamically suitable energy levels. For photoreduction reactions, e.g., hydrogen evolution reaction (HER) or CO_2_ reduction reaction (CO_2_RR), the conduction band (CB) of a COF has to locate at a more negative potential than the equilibrium potential (*E*_equilibrium_) of the reaction, i.e., Δ*E*_red_ = *E*_equilibrium_ – *E*_CB_ > 0 V vs normal hydrogen electrode (NHE). In contrast, for photo-oxidation reactions, such as the oxygen evolution reaction (OER), the valence band (VB) of a COF has to be more positive compared to the *E*_equilibrium_, i.e., Δ*E*_ox_ = *E*_VB_ – *E*_equilibrium_ > 0 V vs NHE. Furthermore, an additional energy difference relative to *E*_equilibrium_, the overpotential (η), is needed to overcome the kinetic barrier of the reaction and realize a certain amount of substrate transformation. η is defined as *E*_applied_ – *E*_equilibrium_, giving negative values for the reduction reactions and positive values for the oxidation reactions. Here, to simplify the discussion, we refer to η as the magnitude of the overpotential, i.e., |η|, and correspondingly, Δ*E*_red_ or Δ*E*_ox_ ≥ η is required for the photocatalytic reaction to proceed. The η value of the fuel-forming reaction is strongly related to the sluggish reaction kinetics. For example, CO_2_RR and OER generally require a larger η than HER.^23^ Accordingly, energy levels of the COF photocatalysts should be designed and tailored toward the specific application. In addition, to obtain a high photocatalytic efficiency, COF photocatalysts should be designed to achieve high ϕ_gen_, by enhancing the light harvesting ability with the use of linkers having large molar extinction coefficients and narrower band gaps, as well as high ϕ_sep_ and ϕ_trans_ by improving charge separation, facilitating charge transport and increasing charge carrier lifetimes, etc. Recent reports have reviewed the progress on COF photocatalyst design for improving solar-to-fuel conversion efficiency.^24−26^

In parallel with optimizing the semiconducting properties of COF photocatalysts, increasing attention has been given to engineering catalytically active sites in recent years. In this regard, constructing COF SSPCs by using COFs as photoabsorber in combination with a single-site catalyst is particularly relevant. On the one hand, single-site catalysts offer the opportunity to make full use of the large surface area of COFs (typically 800 m^2^ g^–1^ to 2000 m^2^ g^–1^), thus increasing photogenerated charge-to-fuel conversion efficiency. On the other hand, considering the fact that single-site catalysts are well-defined and structurally identical, provided they have the same coordination environment, SSPCs afford ideal model systems for studying the relationship between the photocatalytic activity and the physicochemical properties of COFs, such as band energy levels, conjugation, etc.

Since a great number of COFs have been demonstrated to be successful for solar fuel production, a note to clarify which COF systems can be categorized as SSPCs is in place here, given that single-site catalysis is a well-defined terminology. Note that another term conceptually related to single-site catalysts is single-atom heterogeneous catalysts. The latter concept aims to maximize atom-utilization efficiency and refers to strictly individual (i.e., single atom), isolated catalytically active atoms on appropriate supports.^27^ The catalytically active species of single-atom heterogeneous catalysts are isolated single atoms, while single-site catalysts can be atoms, ions, molecular complexes, and clusters, as mentioned above. In addition, single-atom catalysts do not require the support of the isolated atoms having an identical coordination environment, which is distinct from the concept of single-site catalysts.^28^ Indeed, the boundary between single-site and single-atom catalysts is somewhat vague. When all catalytically active atoms are anchored on the support in the same manner and behave identically, single-atom catalysts can also be considered as single-site catalysts.^28^ Therefore, photocatalysts defined by this specific kind of single-atom catalyst can also be categorized as SSPCs.

Two-dimensional (2D) metalated porphyrinic COFs can drive photocatalytic solar fuel production, due to the combination of a catalytic metal center and strong light absorption.^29^ However, considering the short interlayer distance (4–7 Å) of 2D metalated porphyrinic COFs, there are concerns about the accessibility of the metal atoms buried between layers. In addition, the close distance between the metal sites along the stacking direction makes the concept of site isolation debatable. Since both features—accessibility of the catalytic site and site isolation—are key requirements for single-site catalysts, we have not included them as SSPCs in the present Perspective. In addition, we notice that several metal-free COFs have been reported as photocatalysts for solar fuel production. Nevertheless, we refrain from including metal-free COF systems as SSPCs. The primary reason is that trace amounts of metal impurities—from the linker or COF synthesis—can act as the active species, as has been revealed in the context of organic polymer-based photocatalysts.^30,31^ Along similar lines, several papers report dye-sensitized COF photocatalysts in which the COF chromophore itself does not contribute to charge generation.^32−34^ Since light harvesting ability is a crucial property that differentiates COFs from other inorganic porous supports, relying on the light absorption of a dye will discard one of the most important features of COFs as SSPC, which is why dye-sensitized COF photocatalysts are excluded from the current Perspective.

Following the terminology of single-site catalysts, we next discuss the characteristics that COF SSPCs should possess. First, COF SSPCs need to contain spatially isolated and well-defined active sites, which are identical in terms of their coordination environment and are well accessible to reactants. The latter feature clearly benefits from the porosity and high surface area of the COFs. Second, the light absorption of COFs should contribute to charge generation and solar fuel production. Third, it has been found that molecular catalysts may not always retain their integrity and can aggregate or transform into nanoparticles under certain experimental conditions.^35,36^ Hence, great care should be taken to investigate whether *in situ* structural transformation of the cocatalyst occurs during the photocatalytic experiments and whether the true catalyst follows the concept of single-site catalysts.

**Figure 2:**
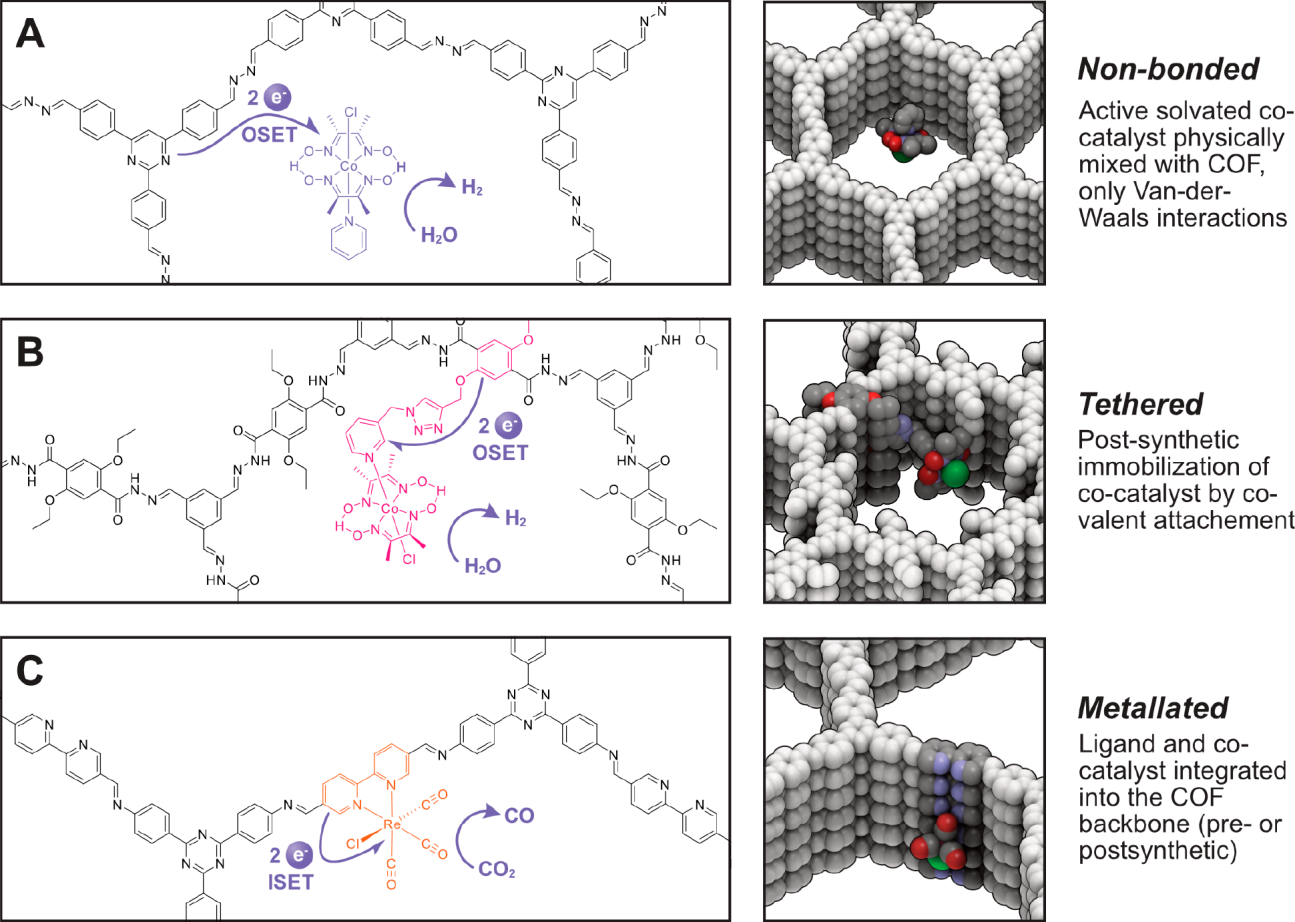
Three main categories of COF SSPCs: non-bonded (A), covalently tethered (B), and metalated COF SSPCs (C). Outer-sphere electron transfer (OSET) is dominant in COF SSPCs based on non-bonded and covalently tethered COF SSPCs, while inner-sphere electron transfer (ISET) is operative in metalated COF SSPCs. Details are described in the text.

COF SSPCs can be classified into three principal categories according to the approach of how the single-site catalyst is introduced into the COFs, as illustrated in Figure 2. Among them, the simplest approach to build COF SSPCs is by dissolving a molecular catalyst in the reaction medium and entrapping it within the COF pores, which provides single-site homogeneous catalyst-based COF SSPCs without chemical bonding between the COF and the cocatalyst (Figure 2A). Under optimal conditions, outer-sphere electron transfer (OSET) takes place between the COF backbone and the molecular catalyst located in the COF pore. A second strategy to build COF SSPCs is by tethering a cocatalyst to the COF, i.e., the covalent anchoring of the cocatalyst to the COF backbone, typically but not necessarily via postsynthetic routes (Figure 2B). The electron transfer mechanism of such COF SSPCs typically remains an OSET, since no direct electronic metal–ligand interaction exists between the metal atom and the COF backbone. However, COF SSPCs can also be obtained by employing an organic ligand as the COF building block, i.e., by direct integration of the catalyst into the COF backbone (Figure 2C). This strategy, in principle, allows for an inner-sphere electron transfer (ISET) between the COF and the cocatalyst. Metal active sites can either be incorporated via postsynthetic metalation of the ligand containing COF, or preloaded into the building block by metalation before COF synthesis. For metalated COF SSPCs, 2,2′-bipyridine (bpy) and its derivatives are the most commonly used building blocks, and various bpy-based COF SSPCs have been reported so far.^37,38^ Based on these concepts, we will now proceed to review representative COF SSPCs for solar fuel production, including solar-driven water splitting and CO_2_ reduction, in the next sections.

## Solar-Driven Water Splitting

Producing hydrogen by photocatalytic water splitting is a promising strategy to meet the target of converting solar energy to a potentially scalable and economically feasible sustainable energy form. COFs are emerging photocatalysts for solar-driven water splitting. In 2014, our group reported COFs as hydrogen evolution photocatalysts for the first time, using a hydrazone-based COF as photoabsorber and Pt as a hydrogen evolution catalyst.^39^ Subsequently, while a rapidly growing number of COFs are found to be photoabsorbers for solar hydrogen evolution, metallic nanoparticulate Pt remains the key hydrogen evolution cocatalyst in the field. Nevertheless, nanoparticulate Pt with nonuniform size and unclear surface faceting not only has low metal atom utilization as only the surface atoms are catalytically active; it also complicates the study of the influence of Pt loading, location, and the specific nature of the active site on photocatalytic activity. Therefore, more and more attention has been devoted to the rational development of single-site hydrogen evolution cocatalysts for COFs, aiming to improve the utilization efficiency of catalytically active atoms, which is particularly important for scarce and costly noble metal atoms.

**Figure 3:**
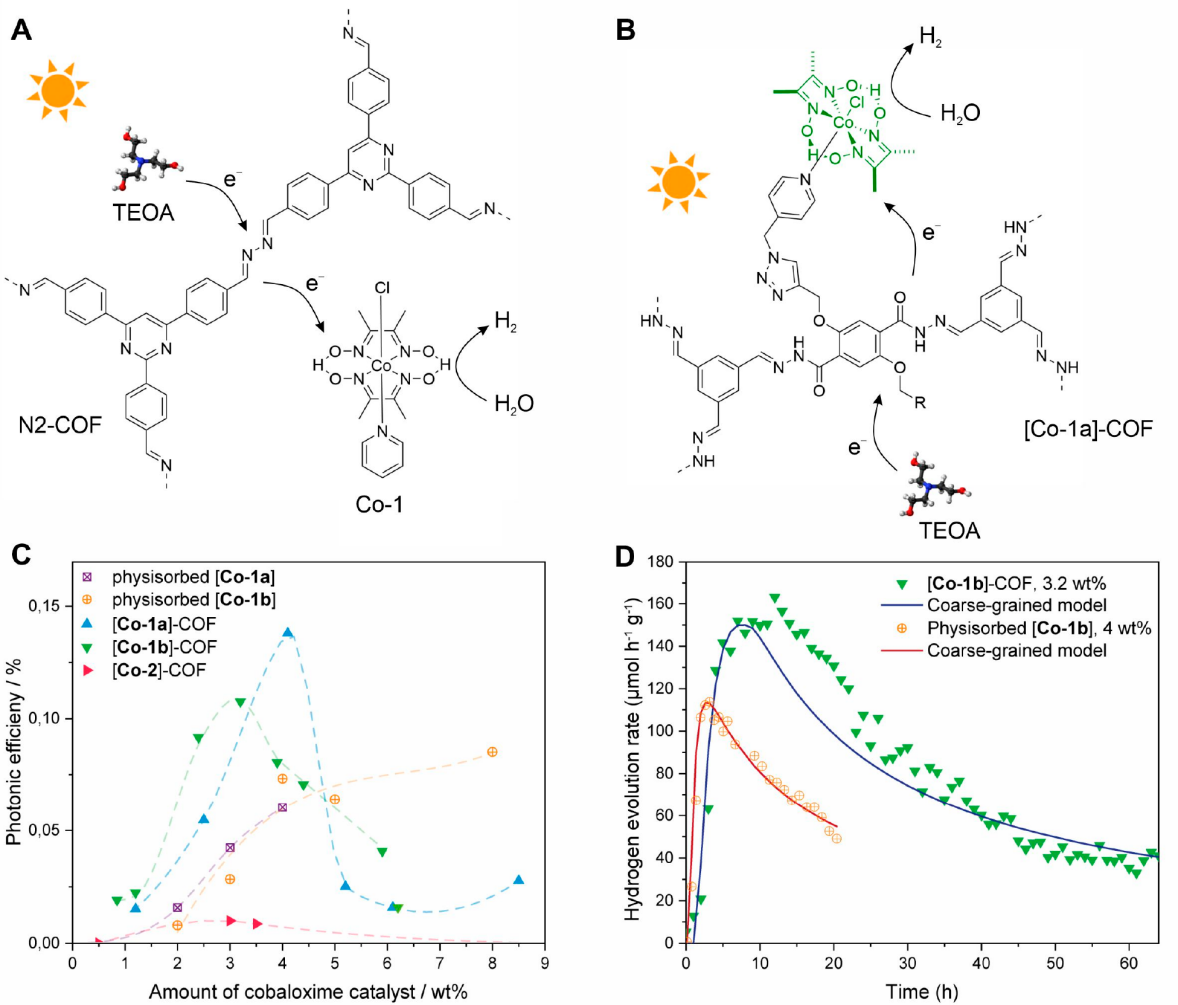
Cobaloxime HER catalyst-based COF SSPC systems. (A) Schematic representation of non-bonded N2-COF/Co-1 SSPC system. Reproduced from ref (40). Copyright 2017 American Chemical Society. (B) Schematic representation of covalently tethered [Co-1a]–COF SSPC. (C) Comparison of photonic efficiencies for a catalyst-tethered system and COF-42 with physisorbed [Co-1a] and [Co-1b]. (D) Comparison of the hydrogen evolution rate of [Co-1b]–COF containing 3.2 wt % [Co-1b] and COF-42 with 4.0 wt % physisorbed [Co-1b] and coarse-grained model fits of both systems. Panels (B–D) are reproduced from ref (41). Copyright 2020 American Chemical Society.

The first COF SSPC demonstration which enabled photocatalytic solar-to-fuel conversion was reported by Banerjee et al. with a non-bonded system (Figure 3A).^40^ In this work, one member of the previously reported N_*x*_-COF series was employed as the photoabsorber, after it had already been established as an efficient photocatalyst via decoration with metallic Pt particles.^42^ Three cobaloxime-based molecular catalysts (Co-1, Co-2, and Co-3) were compared as HER cocatalysts in the COF SSPC system. After systematic optimizations of the photocatalytic conditions, a hydrogen evolution rate of 782 μmol g^–1^ h^–1^ and a turnover number (TON) of 54.4 were achieved for N2-COF/Co-1 in a 4:1 acetonitrile/H_2_O mixture and in the presence of triethanolamine (TEOA) as the sacrificial electron donor (SED). Without any of the three components, N2-COF, TEOA, or Co-1, only negligible amounts of H_2_ were produced. Together with the fact that no trace of cobalt oxide or metallic cobalt was seen after photocatalysis and Co-1 is an established molecular HER catalyst, this result indicates the single-site nature of the COF SSPC system.^35,43^ Control experiments were carried out to exclude the possibility of hydrogen evolution originating from decomposition of the photoabsorber, cobaloxime ligand, or sacrificial agent. By postphotocatalysis characterization of the N2-COF with ^13^C cross-polarization magic angle spinning NMR, FT-IR, and TEM, it was shown that indeed no chemical interaction between N2-COF and cobaloxime molecular catalyst exists, suggesting that an outer-sphere electron transfer drives the N2-COF/Co-1 SSPC system. The authors noted that while N2-COF exhibits excellent chemical stability under photocatalytic operational conditions, the long-term stability of N2-COF/cobaloxime SSPC is a limiting factor, primarily due to the poor photostability of the cobaloxime molecular cocatalyst.

Gottschling et al. further extended the COF/cobaloxime SSPCs by a catalyst-tethered strategy (Figure 3B).^41^ Through postsynthetic click-chemistry, azide-functionalized cobaloximes (Co-1a and Co-1b) were covalently tethered to the alkyne-containing COF-42 backbone, which resulted in catalyst-tethered COF SSPCs, [Co-1a]–COF and [Co-1b]–COF. To analyze the structure of cobaloxime-tethered COFs, 2D solid-state NMR characterization and quantum chemical NMR calculations were performed, suggesting that the cobaloxime in [Co-1a]–COF closely interacts with the pore wall. The photocatalytic hydrogen evolution activity of [Co-1a]–COF and [Co-1b]–COF was measured in an acetonitrile/water mixture with TEOA as SED, which indicated that immobilizing cobaloxime on the COF enhanced the hydrogen evolution rate by more than 100%, compared to mixing the corresponding amount of homogeneous cobaloxime catalyst with COF-42. More importantly, due to the covalent attachment of the cobaloxime cocatalyst, 80% of the initial activity was maintained after 20 h continuous test, while the activity of the homogeneous cobaloxime-based system decreased to 52%. The enhanced activity and stability are related to the local confinement of the cobaloxime cocatalyst in the COF pore, which likely not only improves the electron transfer kinetics from the COF backbone to cobaloxime but also strengthens recoordination of the labile axial pyridine ligand, which otherwise is a key degradation channel of the molecular catalyst. Thus, leaching of the metal–organic catalyst is prevented.

**Figure 4:**
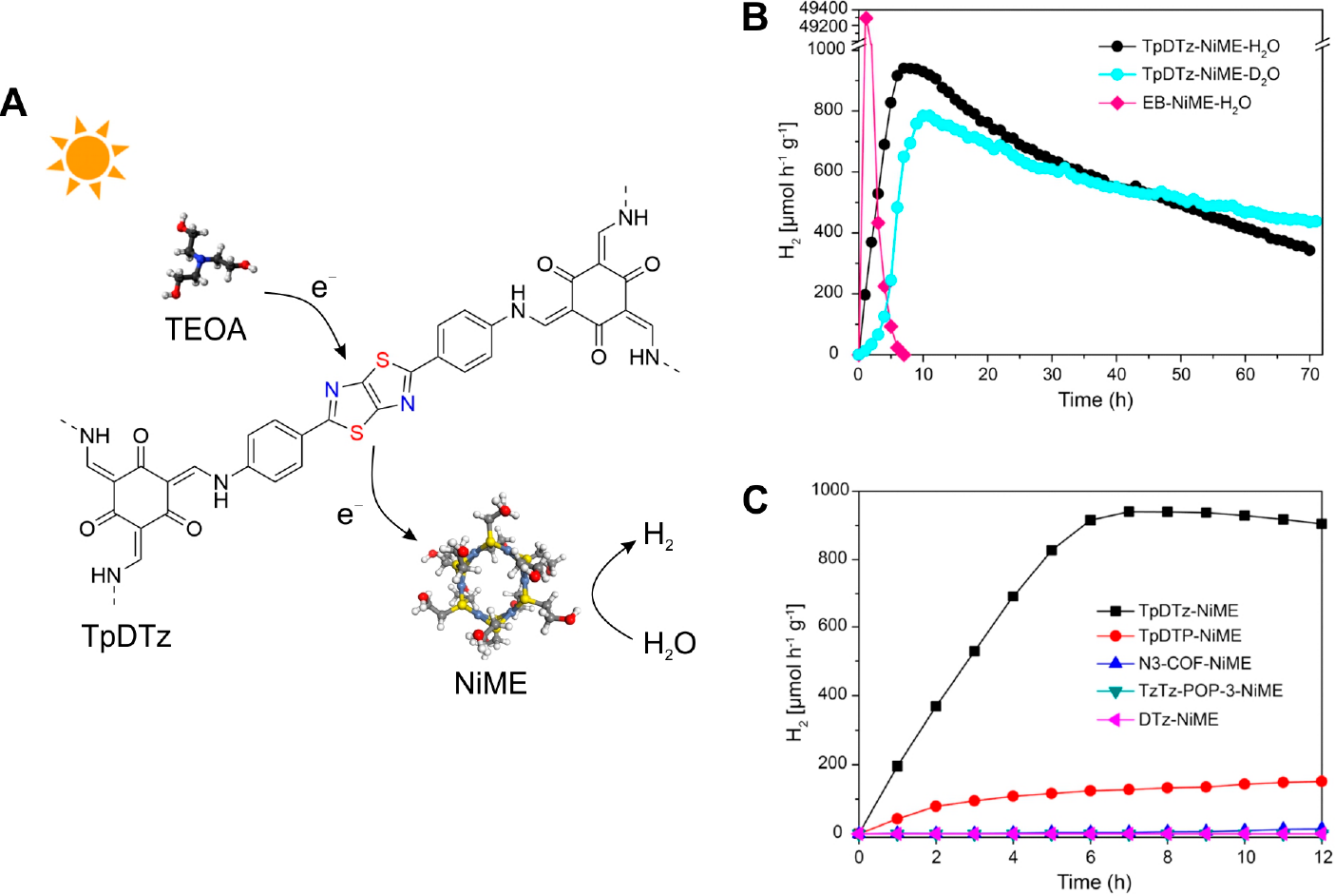
Non-bonded TpDTz/NiME SSPC system. (A) Schematic representation of the building blocks, catalyst, and sacrificial agent and their interplay. (B) Comparison of photocatalytic H_2_ evolution rates in water (H_2_O) and deuterium oxide (D_2_O), using TpDTz COF over 72 h and Erythrosin B dye under AM 1.5 light irradiation. (C) Photocatalytic H_2_ evolution from water using different photosensitizers. Reproduced from ref (44). Copyright 2022 American Chemical Society.

While hydrogen evolution was successfully achieved in the aforementioned COF/cobaloxime SSPCs, a mixed aqueous–organic solvent was required as the reaction medium. The ultimate goal of photocatalytic solar-to-fuel conversion is to perform the reaction in pure water, a green solvent, which naturally contains the highest substrate concentration (i.e., protons), is environmentally benign, and mitigates any unwanted byproducts incurred through the use of organic solvents. Since most of the molecular catalysts can only be operated in organic media or mixed aqueous–organic media, COF SSPCs operating in aqueous media remain challenging. Toward addressing this challenge, Biswal et al. demonstrated a non-bonded COF SSPC, comprising a newly designed thiazolo[5,4-*d*]thiazole-linked COF (TpDTz) as the photoabsorber and a molecular Ni-thiolate cluster (NiME)—assembled *in situ*—as hydrogen evolution catalyst (Figure 4).^44^ In the presence of water as the reaction medium and TEOA as the SED, the optimized TpDTz/NiME SSPC evolved hydrogen with a maximum hydrogen evolution rate of 941 μmol g^–1^ h^–1^ under standard 1 sun illumination. More importantly, the TpDTz/NiME SSPC exhibited excellent stability: continuous hydrogen evolution was observed over a period of at least 70 h, with ∼40% of the initial hydrogen evolution rate being maintained after 70 h. In sharp contrast, Erythrosin B, a common dye for dye-sensitized photocatalytic hydrogen evolution, showed a high initial activity (49297 μmol g^–1^ h^–1^), followed by a rapid decay in hydrogen evolution rate under identical conditions and turned inactive within 7 h. Through investigating the structure–property–activity relationship of a series of samples, the authors noted that both the crystalline structure of TpDTz and the thiazolo[5,4-*d*]thiazole (DTz) building block play critical roles in obtaining the high hydrogen evolution rate: TzTz-POP-3, a DTz based amorphous porous organic polymer, and DTz, the diamine linker, are inactive under the same conditions, while 4,4′′-diamino-*p*-terphenyl based TpDTP exhibits a hydrogen evolution rate of only 160 μmol g^–1^ h^–1^. This observation suggests a synergistic interplay between the larger surface area, smaller optical band gap and better dispersibility of TpDTz in water, and accordingly, underlines the feasibility to reach higher activities by rationally engineering the COF backbone.

Another example of a water-compatible COF SSPC was reported by Dong et al., where atomically dispersed Pt is grafted onto a β-ketoenamine-linked COF, TpPa-1-COF.^45^ Through a molecular organization approach developed by Karak et al.,^46^ TpPa-1-COF/Pt SSPC was synthesized by adding H_2_PtCl_6_·6H_2_O solution into the precursor of TpPa-1-COF and transforming it to TpPa-1-COF/Pt SSPC with subsequent grinding and heating. Benefiting from the preloading of Pt in TpPa-1-COF precursor, Pt atoms were assumed to be confined in the COF pores, leading to a discrete distribution of Pt in TpPa-1-COF at optimal Pt loading (3% Pt_1_@TpPa-1) as well as a higher overall Pt loading amount. In comparison, direct Pt photodeposition from H_2_PtCl_6_·6H_2_O on the as-prepared TpPa-1-COF resulted in only decoration of the COF with Pt nanoparticles (3% Pt NPs/TpPa-1). The isolated nature of the Pt sites in TpPa-1-COF was inferred by transmission electron microscopy (TEM) and extended X-ray absorption fine structure (EXAFS) spectroscopy. Close inspection of the EXAFS data afforded quantitative structural information about the Pt coordination number, suggesting the local Pt coordination environment corresponds to the C_3_N–Pt–Cl_2_ motif in 3% Pt_1_@TpPa-1. In a PBS buffer solution with sodium ascorbate as SED, the optimized 3% Pt_1_@TpPa-1 shows a hydrogen evolution rate of 719 μmol g^–1^ h^–1^ over 6 h of irradiation, 3.9 times higher than 3% Pt NPs/TpPa-1. DFT calculations suggested that the higher activity of TpPa-1-COF/Pt SSPC can be attributed to six-coordinated Pt single atoms, which reduces the energy barrier for the formation of H* on the surface/interface. It is worth mentioning that the authors explicitly described their materials as single-atom catalyst based photocatalysts. Nevertheless, 3% Pt_1_@TpPa-1 also meets the concept of SSPC,^28^ since the Pt active sites are well-defined as suggested by EXAFS analysis and spatially separated, enabling a similar functionality.

Despite the success of COF SSPCs in photocatalytic hydrogen evolution, water oxidation photocatalysts supporting oxygen evolution are required to achieve overall solar water splitting. However, since the complex four-electron transfer process of oxygen formation leads to particularly sluggish reaction kinetics, it remains challenging to develop viable COF SSPCs active for oxygen evolution, even though a certain amount of COFs are expected to be thermodynamically capable of oxidizing water and evolving oxygen.^47^ For example, Chen et al. demonstrated a Co^2+^ functionalized bpy-COF (BpCo-COF-1), which enabled photocatalytic oxygen evolution in the presence of AgNO_3_ as sacrificial electron acceptor (SEA).^48^ Loading 1 wt % Co^2+^ onto the COF resulted in an optimal oxygen evolution rate of 152 μmol g^–1^ h^–1^, while higher Co^2+^ loadings resulted in a decreased activity. Interestingly, a similar COF—based on Tp and Co^2+^ bpy as building blocks—was recently reported to be a catalyst for electrochemical oxygen evolution.^49,50^ Nevertheless, despite the promising potential of Co^2+^ bpy-COFs in photocatalytic/electrocatalytic oxygen evolution, caution is warranted when interpreting such data as cobalt ions or complexes in alkaline media are known to transform to cobalt (oxy)hydroxide/oxides, which are considered as the true active species for oxygen evolution reaction.^51,52^ Thus, further investigations toward identifying the true active species in Co^2+^ and even other metal-based bpy COF catalysts will be necessary in order to establish if these metalated COFs are true single-site (photo)catalysts.

## Solar-Driven CO_2_ Reduction

**Figure 5:**
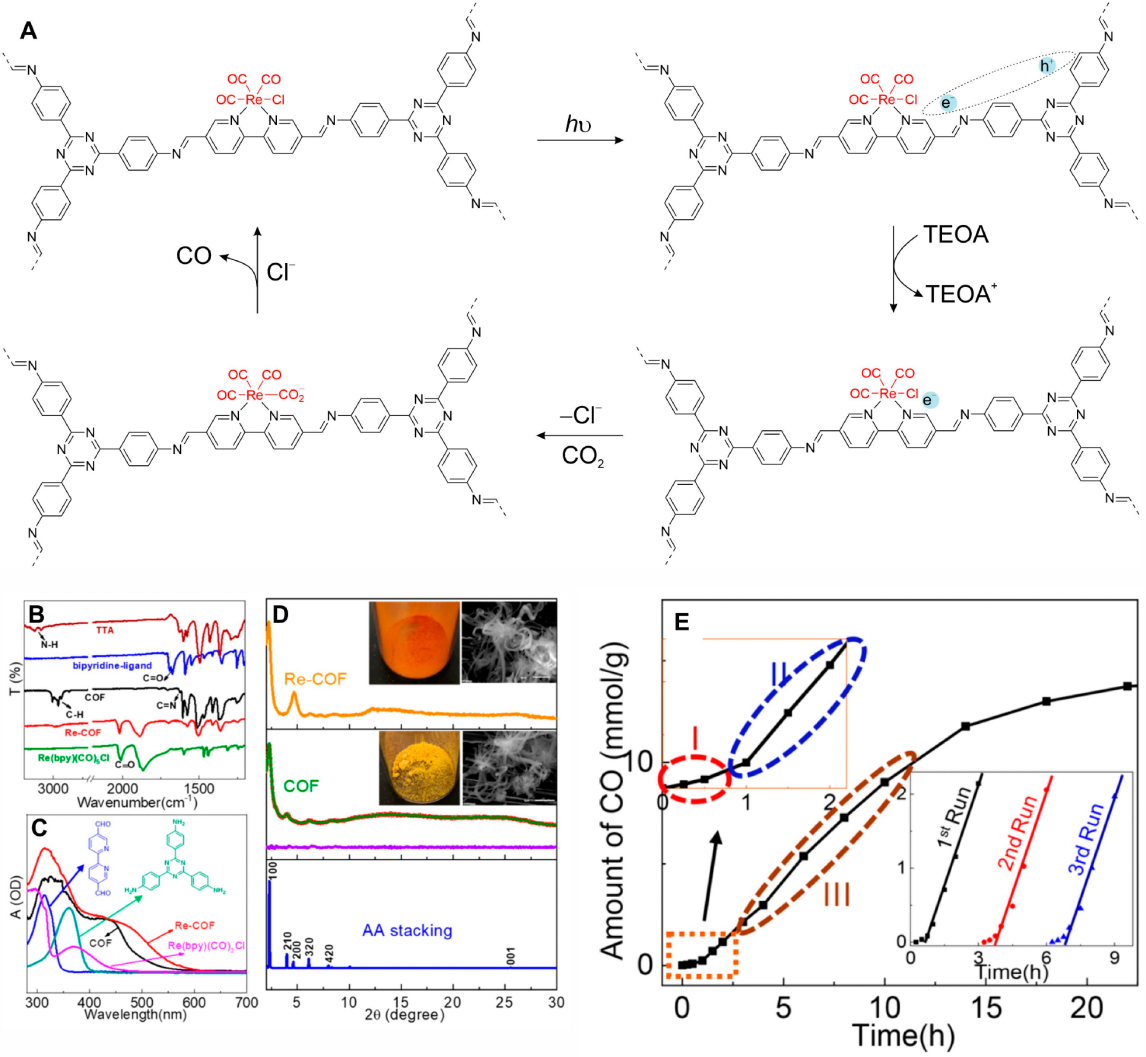
Re-COF SSPC for photocatalytic CO_2_ reduction. Proposed mechanism (A), FT-IR (B), diffuse reflectance UV–visible spectra (C), and powder XRD patterns of Re-COF and COF (D). Amount of CO produced as a function of time (E). Reproduced from ref (37). Copyright 2018 American Chemical Society.

Converting CO_2_ to energy-rich, value-added fuels via photocatalytic reactions offers a sustainable way to recycle CO_2_ while providing an alternative energy source to fossil fuels. While early research on photocatalytic CO_2_ reduction dates back to the 1980s, efforts intensified in the last years due to the pressing demand to reduce greenhouse gas accumulation but also as a consequence of the generated insights from the conceptually simpler HER photocatalysis. Molecular catalysts allow for fine-tuning of chemical and electronic structures as well as high CO_2_ conversion efficiency and selectivity, and have thus been recognized as among the most competitive catalyst candidates for CO_2_ reduction. Therefore, significant efforts have been devoted to developing molecular-catalyst-based COF SSPCs for photocatalytic CO_2_ reduction. The Re^I^(bpy)(CO)_3_X catalyst family—also known as Lehn’s catalyst—where bpy denotes 2,2′-bipyridine and its derivatives and X is an axial ligand such as Cl or Br, has been intensively investigated as CO_2_ reduction photocatalysts since the first report in 1983, given their high selectivity over the generally competing H_2_ formation.^53,54^ In 2018, Yang et al. incorporated Re(bpy)(CO)_3_Cl into an imine-linked bipyridine and triazine COF, Re-COF, providing the first COF SSPC for photocatalytic CO_2_ reduction (Figure 5).^37^ The successful incorporation of the Re complex was confirmed by the appearance of the CO bands in the FT-IR spectrum and by X-ray absorption spectroscopy at the Re L_3_-edge. PXRD and N_2_ physisorption indicated that Re-COF retained the crystallinity and porosity of pristine COF, thus providing a high surface area for photocatalytic reactions. In addition to acting as a catalytically active site, the Re complex also retarded charge recombination in the COF as evidenced by the longer lifetimes measured by transient absorption spectroscopy. Under the illumination with a Xe lamp (225 W, >420 nm) and with TEOA as SED, Re-COF produced ∼15 mmol g^–1^ of CO steadily for >20 h with a CO selectivity of 98% over H_2_. An isotope experiment using ^13^CO_2_ as the reactant showed ^13^CO as the product, confirming that the CO product is indeed obtained from CO_2_ rather than other carbon sources. Moreover, the activity of Re-COF persisted for at least three recycling experiments, showing the potential for practical applications.

**Figure 6:**
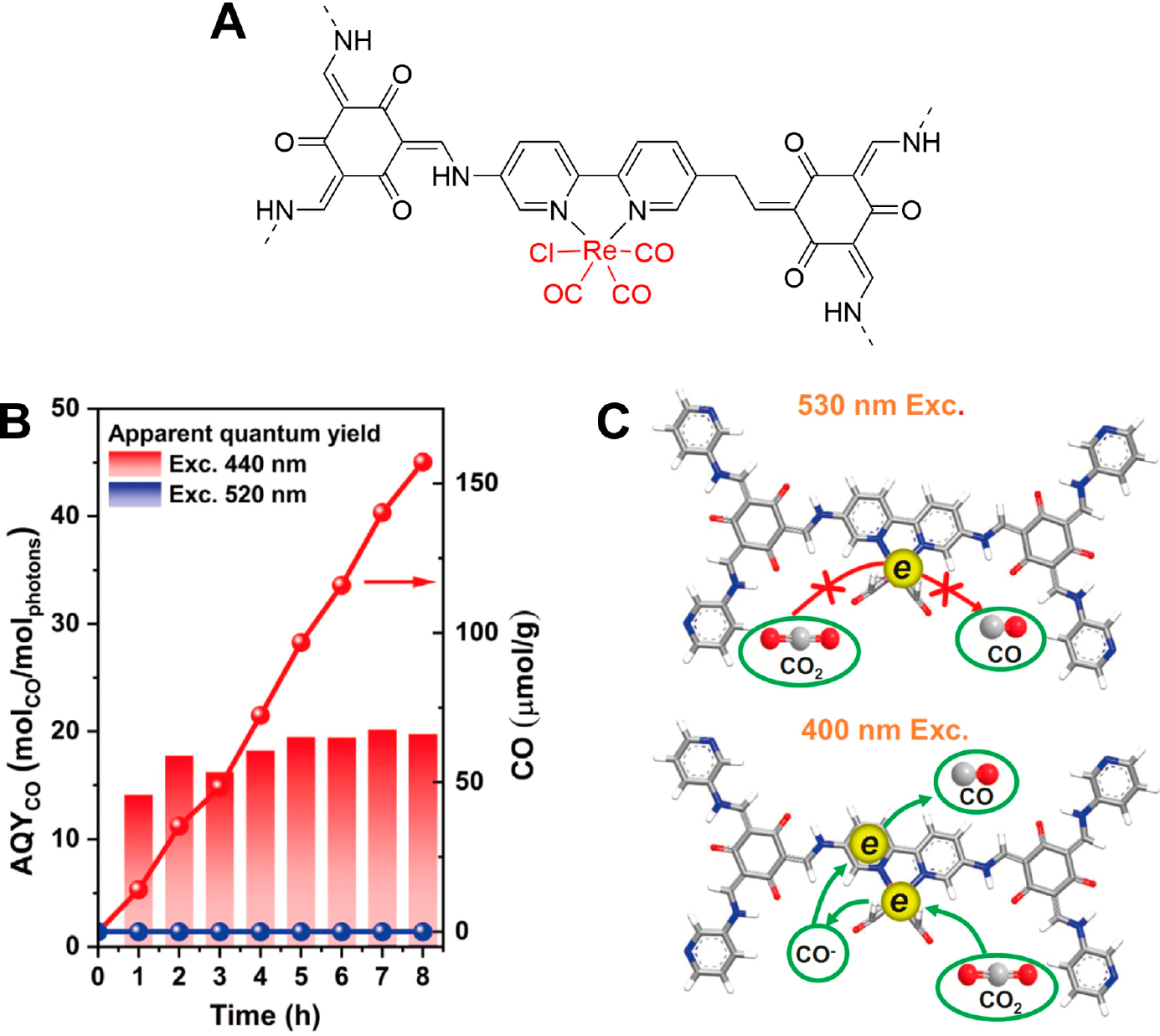
Re-TpBpy SSPC system. Schematic representation of photocatalytic CO_2_ reduction (A). Photocatalytic evolution of CO by Re-TpBpy under 520 and 440 nm excitation (B) and schematic diagram to rationalize the catalytic performance under two excitation conditions (C). Adapted with permission under a Creative Commons CC BY from ref (56). Copyright 2022 Springer Nature.

A β-ketoenamine-linked bipyridine COF, Re-TpBpy, was also used as a photocatalytic CO_2_ reduction SSPC after incorporating a Re complex.^55^ Excited state dynamics of TpBpy and Re-TpBpy were investigated by transient optical spectroscopies, and the results indicated that the introduction of the Re complex into TpBpy facilitated charge separation.^56^ More interestingly, it was found that the activity of Re-TpBpy for CO production is dependent on the excitation energy: an apparent quantum yield of 10 to 15% was obtained by excitation at 440 nm, while the apparent quantum yield under 520 nm excitation is almost negligible (Figure 6). This excitation energy-dependent behavior was rationalized by the observation that an efficient high energy-level electron injection occurs under the excitation of high energy photons, and the electron lifetime at Re^I^ is two times higher compared to band-edge excitation. Triggering this excited state pathway could result in more facile electron transfer from Re^I^ to CO_2_. Furthermore, β-ketoenamine-linked COFs were applied to construct non-bonded COF SSPCs. Kim et al. entrapped Re(CO)_5_Cl molecular catalysts in the pore of two benzothiazole based COFs, TTzTp and BTzTp.^57^ TTzTp and BTzTp SSPCs showed a CO production rate of 586 and 1002 μmol g^–1^ h^–1^, respectively, and ∼98% selectivity over H_2_ in acetonitrile with TEOA as a sacrificial electron donor. The activity of COF SSPCs is more durable compared to a molecular catalyst-photosensitizer couple based on the prototypical organic photosensitizer [Ru(bpy)_3_]Cl_2_, as indicated by the contrast between the stable 6 h of CO_2_ conversion of BTzTp SSPC and the quick decay of the molecular [Ru(bpy)_3_]Cl_2_ system after only 2 h.

While imine and β-ketoenamine-linked COFs exhibit promising activity for photocatalytic CO_2_ reduction, Re complex-based COFs with more conjugated linkages, notably the olefin linkage, promise enhanced chemical robustness, light absorption, and electron delocalization. Fu et al. synthesized a sp^2^-carbon conjugated bipyridine COF (Bpy-sp^2^c-COF) by Knoevenagel condensation.^38^ Postfunctionalization with a Re complex resulted in Re-Bpy-sp^2^c-COF, which was tested for photocatalytic CO_2_ reduction. CO_2_ adsorption isotherms indicated that Re-Bpy-sp^2^c-COF has good affinity for CO_2_, as shown by a CO_2_ adsorption capacity of 1.7 mmol g^–1^ at 278 K and 1.1 mmol g^–1^ at 298 K. Photocatalytic experiments with Re-Bpy-sp^2^c-COF were conducted in acetonitrile under illumination of a 300 W Xe lamp (>420 nm) in the presence of TEOA as SED. CO was formed at a rate of 1040 μmol g^–1^ h^–1^ and a selectivity of 81% over H_2_, while the apparent quantum yield of CO production at 420 nm was determined to be 0.5%. In comparison, only trace amounts of CO were detected when Bpy-sp^2^c-COF, without Re complex, was used as the photocatalyst, thus highlighting the crucial role of the Re complex in catalytic CO_2_ reduction. Moreover, an amorphous polymer, Re-Bpy-sp^2^c-P, was also synthesized, which only produced CO with a formation rate of 96 μmol g^–1^ h^–1^ (∼9% of Re-Bpy-sp^2^c-COF’s CO formation rate). The comparison between Re-Bpy-sp^2^c-COF and Re-Bpy-sp^2^c-P demonstrates once more that the crystallinity and porosity of the COFs are beneficial for solar-to-fuel conversion.

It is indisputable that the scarcity of Re is a limitation for the practical application of Re complex based COF SSPCs. Thus, molecular catalysts based on more abundant elements, such as manganese, nickel, and cobalt, have also been explored to construct COF SSPCs. [Mn(bpy)(CO)_3_Br] and its derivatives were developed as alternative high-abundance molecular catalysts for CO_2_ reduction in recent years.^58^ Under suitable conditions, manganese carbonyl catalysts showed competitive CO_2_ reduction selectivity over H_2_, even in an aqueous medium. Recently, Wang et al. incorporated a manganese carbonyl complex in a triazine-bipyridine based COF and reported the photocatalytic CO_2_ reduction behavior of the resulting Mn-TTA-COF.^59^ Photocatalytic tests in acetonitrile with TEOA as SED indicated that Mn-TTA-COF is capable of a more stable CO production compared to the homogeneous system. Nevertheless, CO production was also observed under a N_2_ atmosphere, implying the decomposition of the manganese carbonyl complex under illumination. Since manganese carbonyl complexes usually exhibit better CO_2_ reduction activity with the assistance of Brønsted acids,^58^ it is expected that there is still room for further improvement by optimizing photocatalytic operation conditions.

**Figure 7:**
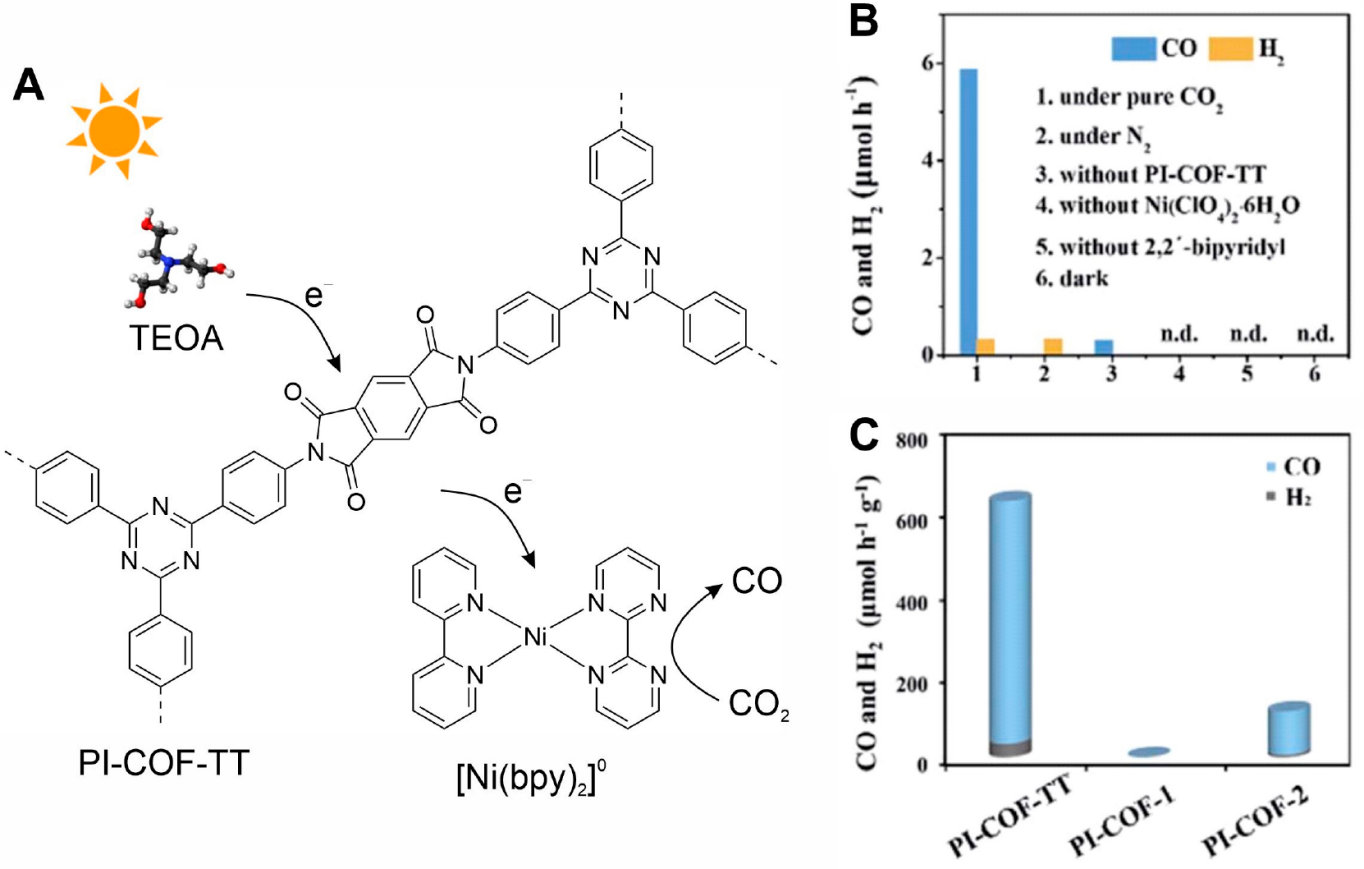
PI-COF-TT SSPC system. Schematic representation of the COF building blocks, catalyst, and SED and the expected electron transfer pathway during photocatalytic CO_2_ reduction (A). Catalytic performance of PI-COFs (B). Control experiments used PI-COF-TT for 2 h of CO_2_ photoreduction (C). Adapted from ref (60) under a Creative Commons Attribution 3.0 Unported License. Copyright 2020 Royal Society of Chemistry.

The Ni molecular catalyst, [Ni(bpy_3_)]^2+^, was also integrated into COFs to construct a noble-metal-free system for photocatalytic CO_2_ reduction.^60^ Three imide-linked COFs, PI-COF-TT, PI-COF-1 and PI-COF-2, with pore sizes ranging from 1.5 to 3.5 nm, were synthesized and employed as light-harvesting materials (Figure 7). The [Ni(bpy_3_)]^2+^ catalyst was formed in the COF pore channels by in situ assembly from the precursors. Interestingly, in situ catalyst formation offers a higher catalytic activity compared to the direct impregnation of [Ni(bpy_3_)]^2+^, likely due to the inefficient diffusion of the Ni complex into the COF pores. The authors proposed that [Ni(bpy_3_)]^2+^ can be converted to [Ni(bpy)_2_]^0^ under illumination and the presence of a SED, and that the latter Ni complex acts as the active species for the CO_2_ reduction reaction. Among the three COFs, PI-COF-TT showed the highest performance with a CO production rate of 1933 μmol g^–1^ and a selectivity of 93% over H_2_. It is noteworthy that a color change of the COF photoabsorber from yellow to blue was observed under photocatalytic operation conditions, while the original color can be recovered upon the exposure of air. Indeed, such a photochromic effect has been reported in several examples where COFs or carbon nitride were employed as photosensitizers. The formation of anion radicals by trapping photogenerated electrons has been invoked as the origin of this effect.^37,40,61,62^ Photoinduced charge trapping enables solar-powered energy storage and, hence, direct light storage. Following this principle, it has been demonstrated that after switching off the illumination, the stored charges can either be converted into hydrogen upon addition of an HER cocatalyst, or be released in the form of electrical energy, affording “dark photocatalysis” or solar batteries, respectively.^61,62^ Hence, the long-lived radical feature of these systems provides an opportunity to overcome the intermittency of solar irradiation, thus addressing the main limitation of solar technologies.

Given that the equilibrium potentials of CO_2_ reduction products are very close in energy to that of reducing water to hydrogen, performing photocatalytic CO_2_ reduction in aqueous media will require the catalyst in the COF SSPC system to be highly selective for CO_2_ reduction over HER. Indeed, all the aforementioned systems only work in organic or mixed organic/aqueous solution, while constructing COF SSPCs active in aqueous medium for CO_2_ reduction remains a great challenge. Toward this end, Xiang et al. synthesized sp^2^c-COF_dpy_-Co by introducing Co^2+^ into a bipyridine-based sp^2^ carbon-linked COF, which enabled successful photocatalytic CO_2_ reduction in water.^63^ With TEOA as the SED, sp^2^c-COF_dpy_-Co generated CO with a conversion rate of 0.99 mmol g^–1^ h^–1^ and a selectivity of 81.4% over H_2_. Replacing bipyridine with biphenyl, however, resulted in a CO formation rate of only 0.01 mmol g^–1^ h^–1^. This suggests that Co ions coordinatively immobilized in the bipy unit in sp^2^c-COF_dpy_-Co play a crucial role in the catalytic CO_2_ reduction. Nevertheless, similar to the BpCo-COF-1 system for photocatalytic oxygen evolution, unambiguously establishing the SSPC nature of sp^2^c-COF_dpy_-Co would require further study of the catalytic mechanism and local environment of the active site, since Co^2+^ ions could be transformed into heterogeneous Co species under basic aqueous conditions, thus highlighting once more the need for a detailed understanding of catalyst speciation in single-site systems.

## Outlook and Future Directions

Since the first report of the COF SSPC in 2017, COF SSPCs have transformed into a burgeoning field. Much of its momentum comes from their conceptual similarity to other porous single-site catalyst platforms including silica, zeolites, and MOFs, combined with their intrinsic light harvesting ability and a rich suite of functionalization strategies, making them an exquisitely well-defined yet robust family of catalysts for solar fuel production. So far, COF SSPCs have shown several unique facets, including promising photocatalytic activities, recyclability of the photocatalysts,^38,57,60^ and extended catalytic stability compared to homogeneous systems,^38,57,59^ which warrant future endeavors but also call for further optimization strategies. For example, while several reports confirmed the vital role of crystallinity and porosity of COFs for obtaining high photocatalytic activities,^38,40,44^ more systematic studies are needed to shed light on the interplay between molecular and extended structure, degree of order, level of defects and local structural effects on the catalytic activities. At the same time, the detailed mechanism of interfacial charge transfer between the COF photoabsorber and the molecular catalyst unit is largely obscure, but a key element in understanding and orchestrating light absorption, charge carrier dynamics, and charge transfer at the COF–cocatalyst interface. These aspects are particularly important for kinetically sluggish half-reactions, including the oxygen evolution and CO_2_ reduction reaction, where charge recombination at the COF/cocatalyst interface appears to be a limiting factor. Such insights may not only provide solutions to enhance photocatalytic activity but also afford strategies for modulating selectivity for the desired reactions, such as CO_2_ reduction over hydrogen evolution in the photocatalytic CO_2_ reduction reaction. In addition, efforts are still needed for COF SSPCs to step up toward overall photocatalytic water splitting and water/moisture-driven CO_2_ reduction via either one-step or two-step photoexcitation systems,^4^ where sacrificial agents are no longer needed. The reason is that although sacrificial agents are useful for understanding the reaction mechanisms of half reactions, the reduction/oxidation of sacrificial agents is often an exergonic process (Δ*G* < 0),^64^ indicating that the absorbed solar energies are not stored in the produced fuels. In two-step photoexcitation systems redox shuttles such as IO_3_^–^/I^–^ are recommended as replacements for sacrificial agents and act as electron donors/acceptors.^65−67^ Alternatively, traditional sacrificials should be replaced by value-added ones, such as biomass or microplastics, where hydrogen evolution or CO_2_ reduction goes hand in hand with the valorization or targeted degradation of reductants or oxidants.

Given the above aspects and the practically unlimited combinations of building blocks, as well as the diverse approaches to introduce single-site catalysts, we surmise that the COF SSPC design for solar fuel production is still in its infancy. In this Perspective, we have summarized the reported strategies to construct COF SSPCs and gave examples for their application in solar energy to fuel conversion, including hydrogen and oxygen evolution as well as CO_2_ reduction. To promote future research into COF SSPCs, it is vital, however, to take a step back and critically outline some of the obvious or more subtle pitfalls, as well as possible directions this field can take to fully embrace its future potential.

### Photocatalytic Activity Reporting

It is generally accepted that comparing the photocatalytic activity reported by different groups is impractical, as various photocatalysis setups are used to evaluate activity, differentiated by reactor types, light sources, internal or external irradiation, mass transfer, etc.^4^ All these factors affect photocatalytic performance and contribute to make quantitative comparison of photocatalytic rates challenging across different laboratories.^68,69^ Although increasing the photocatalytic activity of COF SSPCs remains a crucial task, emphasizing higher photocatalytic activities over other papers is inexpedient without providing details of the setup, such as the irradiation spectrum, irradiation intensity, photocatalyst amount, or reactor size. To further strengthen the comparability of photocatalytic activity, on the one hand, standardized evaluation systems should be used to assess the photocatalytic performance based on—wherever possible—indicators that take into account effectively absorbed or scattered light (i.e., internal quantum efficiency or internal quantum yield).^69,70^ On the other hand, given the challenge of quantifying the light that is effectively absorbed by the photocatalyst in a reactor setup, at least apparent quantum yield (AQY) as a function of irradiation wavelength should be determined and reported together with the product evolution rate. When compared to photocatalytic rates or external apparent quantum efficiencies, AQY still offers the possibility to directly compare the number of photons contributing to the photocatalytic reaction, and is relatively less dependent on irradiation conditions.^71^ Moreover, a maximum of control experiments should be carried out to verify that the product is obtained from the photocatalytic process of COF SSPCs, rather than other pathways, such as COF degradation, decomposition of the sacrificial agent, other carbon/nitrogen sources (for CO_2_RR or nitrogen fixation), or impurity effects.^72^ We particularly emphasize the necessity to cautiously report metal-free or cocatalyst free COF SSPCs, since metal contaminations even on the level of 0.1 wt %, which is likely to be introduced during the linker synthesis such as via Pd catalyzed coupling reactions, could act as the cocatalyst in the photocatalytic process.^30,31^

### Single-Site Catalyst Design

As discussed in the general concepts section, the single-site feature of the cocatalyst in COF SSPC should be carefully verified, since single-site catalysis is a well-defined concept. One direct strategy to construct COF SSPCs is to integrate intact molecular catalysts with COFs and provide them with favorable accessibility and site isolation. Additionally, introducing isolated atoms and/or ions via coordination interactions may also be feasible. Nevertheless, detailed characterizations (such as TEM,^73^ X-ray absorption spectroscopy,^74−76^ or pair distribution function analysis^77^) to confirm the site isolation and coordination would be required to establish the true single-site nature of the cocatalyst. Meanwhile, it is crucial to verify the retention of the cocatalyst structure under the reaction conditions, without transformation to other species such as atom clusters or nanoparticles.^78−80^ Given the scalability and long-term sustainability issues, there is an indisputable need to employ pure water as a reaction medium and to develop catalytically active sites based on earth-abundant elements for COF SSPCs. However, a current challenge toward this direction is that a large number of earth-abundant element-based molecular catalysts only work in nonaqueous media or mixed aqueous–organic media.^35^ In recent years, an increasing amount of noble metal-free molecular cocatalysts compatible with fully aqueous solutions have been identified.^81^ In addition, single-site catalyst design should aim to achieve stable COF SSPCs, since long-term stability is a critical factor for the practical application of COF SSPCs. In this regard, COF SSPC systems acquired via postsynthetic chemistry, ligand metalation, or by embedding catalytically active atoms in the linker, will be of particular interest, given their capability to suppress catalyst degradation, as demonstrated by Gottschling et al.^41^ Along these lines, one effective strategy would be to develop molecular cocatalysts with functional groups for postsynthetic COF assembly. The effectiveness of such a concept has already been established in other organic polymeric photocatalysts. For instance, Ma et al. developed various functional Co–quaterpyridine molecular complexes, which can be covalently grafted on a photoabsorber and afford a favorable photocatalytic CO_2_ reduction activity and a high selectivity (>97%) over HER.^82,83^

### COF Photoabsorber Engineering

Further increasing the photocatalytic performance and completing solar-to-fuel conversion without the need of sacrificial agents are the major tasks for COF SSPCs. Besides the design of novel single-site catalysts, innovative strategies for the development of COF photoabsorbers are also required to mitigate both the nongeminate and geminate charge recombination in COFs. To that end, the tuning of COF structures, including building blocks and linkages, has proven to be effective in increasing photocatalytic activity to a certain degree. Likewise, understanding the impact of the COF chemical structure and its electronic communication with the single-site catalyst as exemplified recently by the concept of graphene conjugated catalysis introduced by Surendranath and co-workers, will be key to capitalize on the molecular level tunability of COF SSPCs.^84,85^ Meanwhile, we discuss a few other emergent strategies in polymeric photocatalysts, which should also be considered for designing new COF photoabsorbers. For example, creating organic nanoparticles with donor/acceptor heterojunctions can facilitate charge generation. Our recent study also indicates that a polymer donor/COF heterojunction can positively shift the photocurrent onset potential of a COF HER photocathode,^86^ which further demonstrates the feasibility of introducing heterojunctions to improve the photocatalytic performance of COF SSPCs. In addition, since the interface between the COF and liquid medium plays a vital role in charge separation, tuning the affinity of COFs for liquid media, such as by modifying the hydrophilicity of the COF, could also bring further improvement. Wang et al. have reported sulfone-containing COFs, showing better water wettability and photocatalytic hydrogen evolution activity.^87^ Nevertheless, such a strategy has not been applied to designing COF SSPCs, or to photocatalytic CO_2_ conversion, where in principle the selectivity of CO_2_RR over HER could also be tuned. Lastly, improving the charge carrier diffusion length of the COFs is another promising approach. Conductive COFs have been rapidly developed in recent years.^88^ Increasing the charge transport ability of the COF photoabsorbers could reduce the charge recombination loss during the photocatalytic fuel formation process. It can been foreseen that the development of conductive COFs will bring unique opportunities to this new generation of COF photoabsorbers for single-site photocatalysts, photoelectrocatalysis,^86,89,90^ and beyond.

## References

[ref1] Ma Y.; Wang X.; Jia Y.; Chen X.; Han H.; Li C. Titanium Dioxide-Based Nanomaterials for Photocatalytic Fuel Generations. Chem. Rev. 2014, 114 (19), 9987–10043. 10.1021/cr500008u. 25098384

[ref2] White J. L.; Baruch M. F.; Pander J. E.; Hu Y.; Fortmeyer I. C.; Park J. E.; Zhang T.; Liao K.; Gu J.; Yan Y.; Shaw T. W.; Abelev E.; Bocarsly A. B. Light-Driven Heterogeneous Reduction of Carbon Dioxide: Photocatalysts and Photoelectrodes. Chem. Rev. 2015, 115 (23), 12888–12935. 10.1021/acs.chemrev.5b00370. 26444652

[ref3] Tran P. D.; Wong L. H.; Barber J.; Loo J. S. C. Recent advances in hybrid photocatalysts for solar fuel production. Energy Environ. Sci. 2012, 5 (3), 5902 10.1039/c2ee02849b.

[ref4] Chen S.; Takata T.; Domen K. Particulate photocatalysts for overall water splitting. Nat. Rev. Mater. 2017, 2 (10), 17050 10.1038/natrevmats.2017.50.

[ref5] Nishiyama H.; Yamada T.; Nakabayashi M.; Maehara Y.; Yamaguchi M.; Kuromiya Y.; Nagatsuma Y.; Tokudome H.; Akiyama S.; Watanabe T.; Narushima R.; Okunaka S.; Shibata N.; Takata T.; Hisatomi T.; Domen K. Photocatalytic solar hydrogen production from water on a 100-m2 scale. Nature 2021, 598 (7880), 304–307. 10.1038/s41586-021-03907-3. 34433207

[ref6] Hisatomi T.; Domen K. Reaction systems for solar hydrogen production via water splitting with particulate semiconductor photocatalysts. Nature Catalysis 2019, 2 (5), 387–399. 10.1038/s41929-019-0242-6.

[ref7] Zhang F.; Li C. Solar to Chemical Energy Conversion: Theory and Application; Sugiyama M., Fujii K., Nakamura S.., Eds.; Springer, 2016; pp 299–317.

[ref8] Takanabe K. Photocatalytic Water Splitting: Quantitative Approaches toward Photocatalyst by Design. ACS Catal. 2017, 7 (11), 8006–8022. 10.1021/acscatal.7b02662.

[ref9] Taylor H. S. A theory of the catalytic surface. Proc. R. Soc. London, Ser. A 1925, 108 (745), 105–111.

[ref10] Thomas J. M.; Raja R.; Lewis D. W. Single-Site Heterogeneous Catalysts. Angew. Chem., Int. Ed. 2005, 44 (40), 6456–6482. 10.1002/anie.200462473. 16211650

[ref11] Kaiser S. K.; Chen Z.; Faust Akl D.; Mitchell S.; Pérez-Ramírez J. Single-Atom Catalysts across the Periodic Table. Chem. Rev. 2020, 120 (21), 11703–11809. 10.1021/acs.chemrev.0c00576. 33085890

[ref12] Gates B. C.; Flytzani-Stephanopoulos M.; Dixon D. A.; Katz A. Atomically dispersed supported metal catalysts: perspectives and suggestions for future research. Catalysis Science & Technology 2017, 7 (19), 4259–4275. 10.1039/C7CY00881C.

[ref13] Che M.; Mori K.; Yamashita H. Elaboration, characterization and properties of silica-based single-site heterogeneous photocatalysts. Proceedings of the Royal Society A: Mathematical, Physical and Engineering Sciences 2012, 468 (2143), 2113–2128. 10.1098/rspa.2012.0139.

[ref14] Brintzinger H. H.; Fischer D.; Mülhaupt R.; Rieger B.; Waymouth R. M. Stereospecific Olefin Polymerization with Chiral Metallocene Catalysts. Angewandte Chemie International Edition in English 1995, 34 (11), 1143–1170. 10.1002/anie.199511431.

[ref15] Wei X.; Wang K.-X.; Guo X.-X.; Chen J.-S. Single-site photocatalysts with a porous structure. Proceedings of the Royal Society A: Mathematical, Physical and Engineering Sciences 2012, 468 (2143), 2099–2112. 10.1098/rspa.2012.0071.

[ref16] Anpo M.; Thomas J. M. Single-site photocatalytic solids for the decomposition of undesirable molecules. Chem. Commun. 2006, (31), 3273 10.1039/b606738g. 16883411

[ref17] Navlani-García M.; Verma P.; Salinas-Torres D.; Raja R.; Mori K.; Yamashita H. Single-Site Heterogeneous Catalysts and Photocatalysts for Emerging Applications. Advanced Heterogeneous Catalysts Volume 2: Applications at the Single-Atom Scale 2020, 1360, 151–188. 10.1021/bk-2020-1360.ch007.

[ref18] Yamashita H.; Mori K.; Kuwahara Y.; Kamegawa T.; Wen M.; Verma P.; Che M. Single-site and nano-confined photocatalysts designed in porous materials for environmental uses and solar fuels. Chem. Soc. Rev. 2018, 47 (22), 8072–8096. 10.1039/C8CS00341F. 29892768

[ref19] Wen M.; Mori K.; Kuwahara Y.; An T.; Yamashita H. Design of Single-Site Photocatalysts by Using Metal-Organic Frameworks as a Matrix. Chemistry - An Asian Journal 2018, 13 (14), 1767–1779. 10.1002/asia.201800444. 29756680

[ref20] Rogge S. M. J.; Bavykina A.; Hajek J.; Garcia H.; Olivos-Suarez A. I.; Sepúlveda-Escribano A.; Vimont A.; Clet G.; Bazin P.; Kapteijn F.; Daturi M.; Ramos-Fernandez E. V.; Llabrés i Xamena F. X.; Van Speybroeck V.; Gascon J. Metal-organic and covalent organic frameworks as single-site catalysts. Chem. Soc. Rev. 2017, 46 (11), 3134–3184. 10.1039/C7CS00033B. 28338128 PMC5708534

[ref21] Haase F.; Lotsch B. V. Solving the COF trilemma: towards crystalline, stable and functional covalent organic frameworks. Chem. Soc. Rev. 2020, 49 (23), 8469–8500. 10.1039/D0CS01027H. 33155009

[ref22] Geng K.; He T.; Liu R.; Dalapati S.; Tan K. T.; Li Z.; Tao S.; Gong Y.; Jiang Q.; Jiang D. Covalent Organic Frameworks: Design, Synthesis, and Functions. Chem. Rev. 2020, 120, 8814–8933. 10.1021/acs.chemrev.9b00550. 31967791

[ref23] Nitopi S.; Bertheussen E.; Scott S. B.; Liu X.; Engstfeld A. K.; Horch S.; Seger B.; Stephens I. E. L.; Chan K.; Hahn C.; Nørskov J. K.; Jaramillo T. F.; Chorkendorff I. Progress and Perspectives of Electrochemical CO2 Reduction on Copper in Aqueous Electrolyte. Chem. Rev. 2019, 119 (12), 7610–7672. 10.1021/acs.chemrev.8b00705. 31117420

[ref24] Banerjee T.; Podjaski F.; Kröger J.; Biswal B. P.; Lotsch B. V. Polymer photocatalysts for solar-to-chemical energy conversion. Nat. Rev. Mater. 2021, 6 (2), 168–190. 10.1038/s41578-020-00254-z.

[ref25] Banerjee T.; Gottschling K.; Savasci G.; Ochsenfeld C.; Lotsch B. V. H2 Evolution with Covalent Organic Framework Photocatalysts. ACS Energy Lett. 2018, 3 (2), 400–409. 10.1021/acsenergylett.7b01123. 29457140 PMC5809981

[ref26] Wang H.; Wang H.; Wang Z.; Tang L.; Zeng G.; Xu P.; Chen M.; Xiong T.; Zhou C.; Li X.; Huang D.; Zhu Y.; Wang Z.; Tang J. Covalent organic framework photocatalysts: structures and applications. Chem. Soc. Rev. 2020, 49 (12), 4135–4165. 10.1039/D0CS00278J. 32421139

[ref27] Qiao B.; Wang A.; Yang X.; Allard L. F.; Jiang Z.; Cui Y.; Liu J.; Li J.; Zhang T. Single-atom catalysis of CO oxidation using Pt1/FeOx. Nat. Chem. 2011, 3 (8), 634–641. 10.1038/nchem.1095. 21778984

[ref28] Gao C.; Low J.; Long R.; Kong T.; Zhu J.; Xiong Y. Heterogeneous Single-Atom Photocatalysts: Fundamentals and Applications. Chem. Rev. 2020, 120 (21), 12175–12216. 10.1021/acs.chemrev.9b00840. 32186373

[ref29] Gong Y.-N.; Zhong W.; Li Y.; Qiu Y.; Zheng L.; Jiang J.; Jiang H.-L. Regulating Photocatalysis by Spin-State Manipulation of Cobalt in Covalent Organic Frameworks. J. Am. Chem. Soc. 2020, 142 (39), 16723–16731. 10.1021/jacs.0c07206. 32894021

[ref30] Li L.; Cai Z.; Wu Q.; Lo W.-Y.; Zhang N.; Chen L. X.; Yu L. Rational Design of Porous Conjugated Polymers and Roles of Residual Palladium for Photocatalytic Hydrogen Production. J. Am. Chem. Soc. 2016, 138 (24), 7681–7686. 10.1021/jacs.6b03472. 27254306

[ref31] Kosco J.; Sachs M.; Godin R.; Kirkus M.; Francas L.; Bidwell M.; Qureshi M.; Anjum D.; Durrant J. R.; McCulloch I. The Effect of Residual Palladium Catalyst Contamination on the Photocatalytic Hydrogen Evolution Activity of Conjugated Polymers. Adv. Energy Mater. 2018, 8 (34), 1802181 10.1002/aenm.201802181.

[ref32] Wang X.; Fu Z.; Zheng L.; Zhao C.; Wang X.; Chong S. Y.; McBride F.; Raval R.; Bilton M.; Liu L.; Wu X.; Chen L.; Sprick R. S.; Cooper A. I. Covalent Organic Framework Nanosheets Embedding Single Cobalt Sites for Photocatalytic Reduction of Carbon Dioxide. Chem. Mater. 2020, 32 (21), 9107–9114. 10.1021/acs.chemmater.0c01642.

[ref33] Liu W.; Li X.; Wang C.; Pan H.; Liu W.; Wang K.; Zeng Q.; Wang R.; Jiang J. A Scalable General Synthetic Approach toward Ultrathin Imine-Linked Two-Dimensional Covalent Organic Framework Nanosheets for Photocatalytic CO2 Reduction. J. Am. Chem. Soc. 2019, 141 (43), 17431–17440. 10.1021/jacs.9b09502. 31608638

[ref34] Zhong W.; Sa R.; Li L.; He Y.; Li L.; Bi J.; Zhuang Z.; Yu Y.; Zou Z. A Covalent Organic Framework Bearing Single Ni Sites as a Synergistic Photocatalyst for Selective Photoreduction of CO2 to CO. J. Am. Chem. Soc. 2019, 141 (18), 7615–7621. 10.1021/jacs.9b02997. 30998334

[ref35] Zhang B.; Sun L. Artificial photosynthesis: opportunities and challenges of molecular catalysts. Chem. Soc. Rev. 2019, 48 (7), 2216–2264. 10.1039/C8CS00897C. 30895997

[ref36] Li J.; Triana C. A.; Wan W.; Adiyeri Saseendran D. P.; Zhao Y.; Balaghi S. E.; Heidari S.; Patzke G. R. Molecular and heterogeneous water oxidation catalysts: recent progress and joint perspectives. Chem. Soc. Rev. 2021, 50 (4), 2444–2485. 10.1039/D0CS00978D. 33404560

[ref37] Yang S.; Hu W.; Zhang X.; He P.; Pattengale B.; Liu C.; Cendejas M.; Hermans I.; Zhang X.; Zhang J.; Huang J. 2D Covalent Organic Frameworks as Intrinsic Photocatalysts for Visible Light-Driven CO2 Reduction. J. Am. Chem. Soc. 2018, 140 (44), 14614–14618. 10.1021/jacs.8b09705. 30352504

[ref38] Fu Z.; Wang X.; Gardner A. M.; Wang X.; Chong S. Y.; Neri G.; Cowan A. J.; Liu L.; Li X.; Vogel A.; Clowes R.; Bilton M.; Chen L.; Sprick R. S.; Cooper A. I. A stable covalent organic framework for photocatalytic carbon dioxide reduction. Chemical Science 2020, 11 (2), 543–550. 10.1039/C9SC03800K. 32206271 PMC7069507

[ref39] Stegbauer L.; Schwinghammer K.; Lotsch B. V. A hydrazone-based covalent organic framework for photocatalytic hydrogen production. Chem. Sci. 2014, 5 (7), 2789–2793. 10.1039/C4SC00016A. PMC12977080 41822174

[ref40] Banerjee T.; Haase F.; Savasci G.; Gottschling K.; Ochsenfeld C.; Lotsch B. V. Single-Site Photocatalytic H2 Evolution from Covalent Organic Frameworks with Molecular Cobaloxime Co-Catalysts. J. Am. Chem. Soc. 2017, 139 (45), 16228–16234. 10.1021/jacs.7b07489. 29022345 PMC5691321

[ref41] Gottschling K.; Savasci G.; Vignolo-González H.; Schmidt S.; Mauker P.; Banerjee T.; Rovó P.; Ochsenfeld C.; Lotsch B. V. Rational Design of Covalent Cobaloxime-Covalent Organic Framework Hybrids for Enhanced Photocatalytic Hydrogen Evolution. J. Am. Chem. Soc. 2020, 142 (28), 12146–12156. 10.1021/jacs.0c02155. 32564604 PMC7366382

[ref42] Vyas V. S.; Haase F.; Stegbauer L.; Savasci G.; Podjaski F.; Ochsenfeld C.; Lotsch B. V. A tunable azine covalent organic framework platform for visible light-induced hydrogen generation. Nat. Commun. 2015, 6 (1), 8508 10.1038/ncomms9508. 26419805 PMC4598847

[ref43] Dempsey J. L.; Brunschwig B. S.; Winkler J. R.; Gray H. B. Hydrogen Evolution Catalyzed by Cobaloximes. Acc. Chem. Res. 2009, 42 (12), 1995–2004. 10.1021/ar900253e. 19928840

[ref44] Biswal B. P.; Vignolo-González H. A.; Banerjee T.; Grunenberg L.; Savasci G.; Gottschling K.; Nuss J.; Ochsenfeld C.; Lotsch B. V. Sustained Solar H2 Evolution from a Thiazolo[5,4-d]thiazole-Bridged Covalent Organic Framework and Nickel-Thiolate Cluster in Water. J. Am. Chem. Soc. 2019, 141 (28), 11082–11092. 10.1021/jacs.9b03243. 31260279 PMC6646957

[ref45] Dong P.; Wang Y.; Zhang A.; Cheng T.; Xi X.; Zhang J. Platinum Single Atoms Anchored on a Covalent Organic Framework: Boosting Active Sites for Photocatalytic Hydrogen Evolution. ACS Catal. 2021, 11 (21), 13266–13279. 10.1021/acscatal.1c03441.

[ref46] Karak S.; Kandambeth S.; Biswal B. P.; Sasmal H. S.; Kumar S.; Pachfule P.; Banerjee R. Constructing Ultraporous Covalent Organic Frameworks in Seconds via an Organic Terracotta Process. J. Am. Chem. Soc. 2017, 139 (5), 1856–1862. 10.1021/jacs.6b08815. 28106987

[ref47] Saunders B.; Wilbraham L.; Prentice A. W.; Sprick R. S.; Zwijnenburg M. A. The potential scarcity, or not, of polymeric overall water splitting photocatalysts. Sustainable Energy & Fuels 2022, 6 (9), 2233–2242. 10.1039/D2SE00027J.

[ref48] Chen J.; Tao X.; Li C.; Ma Y.; Tao L.; Zheng D.; Zhu J.; Li H.; Li R.; Yang Q. Synthesis of bipyridine-based covalent organic frameworks for visible-light-driven photocatalytic water oxidation. Appl. Catal. B: Environmental 2020, 262, 118271 10.1016/j.apcatb.2019.118271.

[ref49] Aiyappa H. B.; Thote J.; Shinde D. B.; Banerjee R.; Kurungot S. Cobalt-Modified Covalent Organic Framework as a Robust Water Oxidation Electrocatalyst. Chem. Mater. 2016, 28 (12), 4375–4379. 10.1021/acs.chemmater.6b01370.

[ref50] Zhao X.; Pachfule P.; Li S.; Langenhahn T.; Ye M.; Schlesiger C.; Praetz S.; Schmidt J.; Thomas A. Macro/Microporous Covalent Organic Frameworks for Efficient Electrocatalysis. J. Am. Chem. Soc. 2019, 141 (16), 6623–6630. 10.1021/jacs.9b01226. 30916950

[ref51] Hong D.; Jung J.; Park J.; Yamada Y.; Suenobu T.; Lee Y.-M.; Nam W.; Fukuzumi S. Water-soluble mononuclear cobalt complexes with organic ligands acting as precatalysts for efficient photocatalytic water oxidation. Energy Environ. Sci. 2012, 5 (6), 7606 10.1039/c2ee21185h.

[ref52] Hosseini P.; Rodríguez-Camargo A.; Yao L.; Lotsch B.; Tschulik K. Identifying the active species in a cobalt-based covalent organic framework for the electrochemical oxygen evolution reaction. ChemRxiv, September 22, 2023. 10.26434/chemrxiv-2023-7dl21. PMC11744715 39587979

[ref53] Elgrishi N.; Chambers M. B.; Wang X.; Fontecave M. Molecular polypyridine-based metal complexes as catalysts for the reduction of CO2. Chem. Soc. Rev. 2017, 46 (3), 761–796. 10.1039/C5CS00391A. 28084485

[ref54] Windle C. D.; Perutz R. N. Advances in molecular photocatalytic and electrocatalytic CO2 reduction. Coord. Chem. Rev. 2012, 256 (21–22), 2562–2570. 10.1016/j.ccr.2012.03.010.

[ref55] Li S.-Y.; Meng S.; Zou X.; El-Roz M.; Telegeev I.; Thili O.; Liu T. X.; Zhu G. Rhenium-functionalized covalent organic framework photocatalyst for efficient CO2 reduction under visible light. Microporous Mesoporous Mater. 2019, 285, 195–201. 10.1016/j.micromeso.2019.05.026.

[ref56] Pan Q.; Abdellah M.; Cao Y.; Lin W.; Liu Y.; Meng J.; Zhou Q.; Zhao Q.; Yan X.; Li Z.; Cui H.; Cao H.; Fang W.; Tanner D. A.; Abdel-Hafiez M.; Zhou Y.; Pullerits T.; Canton S. E.; Xu H.; Zheng K. Ultrafast charge transfer dynamics in 2D covalent organic frameworks/Re-complex hybrid photocatalyst. Nat. Commun. 2022, 13 (1), 845 10.1038/s41467-022-28409-2. 35149679 PMC8837612

[ref57] Kim Y. H.; Kim N.; Seo J.-M.; Jeon J.-P.; Noh H.-J.; Kweon D. H.; Ryu J.; Baek J.-B. Benzothiazole-Based Covalent Organic Frameworks with Different Symmetrical Combinations for Photocatalytic CO2 Conversion. Chem. Mater. 2021, 33 (22), 8705–8711. 10.1021/acs.chemmater.1c02660.

[ref58] Siritanaratkul B.; Eagle C.; Cowan A. J. Manganese Carbonyl Complexes as Selective Electrocatalysts for CO2 Reduction in Water and Organic Solvents. Acc. Chem. Res. 2022, 55 (7), 955–965. 10.1021/acs.accounts.1c00692. 35285618 PMC9007415

[ref59] Wang D.; Streater D.; Peng Y.; Huang J. 2D Covalent Organic Frameworks with an Incorporated Manganese Complex for Light Driven Carbon Dioxide Reduction. ChemPhotoChem. 2021, 5 (12), 1119–1123. 10.1002/cptc.202100123.

[ref60] Chen X.; Dang Q.; Sa R.; Li L.; Li L.; Bi J.; Zhang Z.; Long J.; Yu Y.; Zou Z. Integrating single Ni sites into biomimetic networks of covalent organic frameworks for selective photoreduction of CO2. Chemical Science 2020, 11 (26), 6915–6922. 10.1039/D0SC01747G. 33033603 PMC7499818

[ref61] Lau V. W.-h.; Klose D.; Kasap H.; Podjaski F.; Pignié M.-C.; Reisner E.; Jeschke G.; Lotsch B. V. Dark Photocatalysis: Storage of Solar Energy in Carbon Nitride for Time-Delayed Hydrogen Generation. Angew. Chem., Int. Ed. 2017, 56 (2), 510–514. 10.1002/anie.201608553. PMC6680103 27930846

[ref62] Podjaski F.; Kröger J.; Lotsch B. V. Toward an Aqueous Solar Battery: Direct Electrochemical Storage of Solar Energy in Carbon Nitrides. Adv. Mater. 2018, 30 (9), 1705477 10.1002/adma.201705477. 29318675

[ref63] Xiang Y.; Dong W.; Wang P.; Wang S.; Ding X.; Ichihara F.; Wang Z.; Wada Y.; Jin S.; Weng Y.; Chen H.; Ye J. Constructing electron delocalization channels in covalent organic frameworks powering CO2 photoreduction in water. Appl. Catal. B: Environmental 2020, 274, 119096 10.1016/j.apcatb.2020.119096.

[ref64] Osterloh F. E. Photocatalysis versus Photosynthesis: A Sensitivity Analysis of Devices for Solar Energy Conversion and Chemical Transformations. ACS Energy Lett. 2017, 2 (2), 445–453. 10.1021/acsenergylett.6b00665.

[ref65] Han L.; Lin M.; Haussener S. Reliable Performance Characterization of Mediated Photocatalytic Water-Splitting Half Reactions. ChemSusChem 2017, 10 (10), 2158–2166. 10.1002/cssc.201601901. 28134489

[ref66] Abe R.; Shinmei K.; Koumura N.; Hara K.; Ohtani B. Visible-Light-Induced Water Splitting Based on Two-Step Photoexcitation between Dye-Sensitized Layered Niobate and Tungsten Oxide Photocatalysts in the Presence of a Triiodide/Iodide Shuttle Redox Mediator. J. Am. Chem. Soc. 2013, 135 (45), 16872–16884. 10.1021/ja4048637. 24128384

[ref67] Abe R.; Sayama K.; Arakawa H. Significant influence of solvent on hydrogen production from aqueous I3-/I- redox solution using dye-sensitized Pt/TiO2 photocatalyst under visible light irradiation. Chem. Phys. Lett. 2003, 379 (3–4), 230–235. 10.1016/j.cplett.2003.07.026.

[ref68] Wang Z.; Hisatomi T.; Li R.; Sayama K.; Liu G.; Domen K.; Li C.; Wang L. Efficiency Accreditation and Testing Protocols for Particulate Photocatalysts toward Solar Fuel Production. Joule 2021, 5 (2), 344–359. 10.1016/j.joule.2021.01.001.

[ref69] Vignolo-González H. A.; Laha S.; Jiménez-Solano A.; Oshima T.; Duppel V.; Schützendübe P.; Lotsch B. V. Toward Standardized Photocatalytic Oxygen Evolution Rates Using RuO2@TiO2 as a Benchmark. Matter 2020, 3 (2), 464–486. 10.1016/j.matt.2020.07.021. 32803152 PMC7418450

[ref70] Qureshi M.; Takanabe K. Insights on Measuring and Reporting Heterogeneous Photocatalysis: Efficiency Definitions and Setup Examples. Chem. Mater. 2017, 29 (1), 158–167. 10.1021/acs.chemmater.6b02907.

[ref71] Wang Z.; Li C.; Domen K. Recent developments in heterogeneous photocatalysts for solar-driven overall water splitting. Chem. Soc. Rev. 2019, 48 (7), 2109–2125. 10.1039/C8CS00542G. 30328438

[ref72] Christopher P.; Jin S.; Sivula K.; Kamat P. V. Why Seeing Is Not Always Believing: Common Pitfalls in Photocatalysis and Electrocatalysis. ACS Energy Lett. 2021, 6 (2), 707–709. 10.1021/acsenergylett.1c00064.

[ref73] Zuo Z.; Liu S.; Wang Z.; Liu C.; Huang W.; Huang J.; Liu P. Dry Reforming of Methane on Single-Site Ni/MgO Catalysts: Importance of Site Confinement. ACS Catal. 2018, 8 (10), 9821–9835. 10.1021/acscatal.8b02277.

[ref74] Timoshenko J.; Roldan Cuenya B. In Situ/Operando Electrocatalyst Characterization by X-ray Absorption Spectroscopy. Chem. Rev. 2021, 121 (2), 882–961. 10.1021/acs.chemrev.0c00396. 32986414 PMC7844833

[ref75] Hoffman A. S.; Debefve L. M.; Zhang S.; Perez-Aguilar J. E.; Conley E. T.; Justl K. R.; Arslan I.; Dixon D. A.; Gates B. C. Beating Heterogeneity of Single-Site Catalysts: MgO-Supported Iridium Complexes. ACS Catal. 2018, 8 (4), 3489–3498. 10.1021/acscatal.8b00143.

[ref76] Zhang H.; Li X.; Jiang Z. Probe active sites of heterogeneous electrocatalysts by X-ray absorption spectroscopy: From single atom to complex multi-element composites. Current Opinion in Electrochemistry 2019, 14, 7–15. 10.1016/j.coelec.2018.09.011.

[ref77] Terban M. W.; Billinge S. J. L. Structural Analysis of Molecular Materials Using the Pair Distribution Function. Chem. Rev. 2022, 122 (1), 1208–1272. 10.1021/acs.chemrev.1c00237. 34788012 PMC8759070

[ref78] Yin Q.; Tan J. M.; Besson C.; Geletii Y. V.; Musaev D. G.; Kuznetsov A. E.; Luo Z.; Hardcastle K. I.; Hill C. L. A Fast Soluble Carbon-Free Molecular Water Oxidation Catalyst Based on Abundant Metals. Science 2010, 328 (5976), 342–345. 10.1126/science.1185372. 20223949

[ref79] Wang J.-W.; Sahoo P.; Lu T.-B. Reinvestigation of Water Oxidation Catalyzed by a Dinuclear Cobalt Polypyridine Complex: Identification of CoOx as a Real Heterogeneous Catalyst. ACS Catal. 2016, 6 (8), 5062–5068. 10.1021/acscatal.6b00798.

[ref80] Kaeffer N.; Morozan A.; Fize J.; Martinez E.; Guetaz L.; Artero V. The Dark Side of Molecular Catalysis: Diimine-Dioxime Cobalt Complexes Are Not the Actual Hydrogen Evolution Electrocatalyst in Acidic Aqueous Solutions. ACS Catal. 2016, 6 (6), 3727–3737. 10.1021/acscatal.6b00378.

[ref81] Dalle K. E.; Warnan J.; Leung J. J.; Reuillard B.; Karmel I. S.; Reisner E. Electro- and Solar-Driven Fuel Synthesis with First Row Transition Metal Complexes. Chem. Rev. 2019, 119 (4), 2752–2875. 10.1021/acs.chemrev.8b00392. 30767519 PMC6396143

[ref82] Ma B.; Blanco M.; Calvillo L.; Chen L.; Chen G.; Lau T.-C.; Dražić G.; Bonin J.; Robert M.; Granozzi G. Hybridization of Molecular and Graphene Materials for CO2 Photocatalytic Reduction with Selectivity Control. J. Am. Chem. Soc. 2021, 143 (22), 8414–8425. 10.1021/jacs.1c02250. 34033471

[ref83] Ma B.; Chen G.; Fave C.; Chen L.; Kuriki R.; Maeda K.; Ishitani O.; Lau T.-C.; Bonin J.; Robert M. Efficient Visible-Light-Driven CO2 Reduction by a Cobalt Molecular Catalyst Covalently Linked to Mesoporous Carbon Nitride. J. Am. Chem. Soc. 2020, 142 (13), 6188–6195. 10.1021/jacs.9b13930. 32148034

[ref84] Jackson M. N.; Kaminsky C. J.; Oh S.; Melville J. F.; Surendranath Y. Graphite Conjugation Eliminates Redox Intermediates in Molecular Electrocatalysis. J. Am. Chem. Soc. 2019, 141 (36), 14160–14167. 10.1021/jacs.9b04981. 31353897 PMC6748662

[ref85] Jackson M. N.; Pegis M. L.; Surendranath Y. Graphite-Conjugated Acids Reveal a Molecular Framework for Proton-Coupled Electron Transfer at Electrode Surfaces. ACS Central Science 2019, 5 (5), 831–841. 10.1021/acscentsci.9b00114. 31139719 PMC6535968

[ref86] Yao L.; Rodríguez-Camargo A.; Xia M.; Mücke D.; Guntermann R.; Liu Y.; Grunenberg L.; Jiménez-Solano A.; Emmerling S. T.; Duppel V.; Sivula K.; Bein T.; Qi H.; Kaiser U.; Grätzel M.; Lotsch B. V. Covalent Organic Framework Nanoplates Enable Solution-Processed Crystalline Nanofilms for Photoelectrochemical Hydrogen Evolution. J. Am. Chem. Soc. 2022, 144 (23), 10291–10300. 10.1021/jacs.2c01433. 35657204 PMC9204765

[ref87] Wang X.; Chen L.; Chong S. Y.; Little M. A.; Wu Y.; Zhu W.-H.; Clowes R.; Yan Y.; Zwijnenburg M. A.; Sprick R. S.; Cooper A. I. Sulfone-containing covalent organic frameworks for photocatalytic hydrogen evolution from water. Nat. Chem. 2018, 10 (12), 1180–1189. 10.1038/s41557-018-0141-5. 30275507

[ref88] Yang Y.; Börjesson K. Electroactive covalent organic frameworks: a new choice for organic electronics. Trends in Chemistry 2022, 4, 60–75. 10.1016/j.trechm.2021.10.007.

[ref89] Sick T.; Hufnagel A. G.; Kampmann J.; Kondofersky I.; Calik M.; Rotter J. M.; Evans A.; Döblinger M.; Herbert S.; Peters K.; Böhm D.; Knochel P.; Medina D. D.; Fattakhova-Rohlfing D.; Bein T. Oriented Films of Conjugated 2D Covalent Organic Frameworks as Photocathodes for Water Splitting. J. Am. Chem. Soc. 2018, 140 (6), 2085–2092. 10.1021/jacs.7b06081. 29249151 PMC6400428

[ref90] Rotter J. M.; Weinberger S.; Kampmann J.; Sick T.; Shalom M.; Bein T.; Medina D. D. Covalent Organic Framework Films through Electrophoretic Deposition—Creating Efficient Morphologies for Catalysis. Chem. Mater. 2019, 31 (24), 10008–10016. 10.1021/acs.chemmater.9b02286.

